# Experimental Convection Heat Transfer Analysis of a Nano-Enhanced Industrial Coolant

**DOI:** 10.3390/nano9020267

**Published:** 2019-02-15

**Authors:** Eva Álvarez-Regueiro, Javier P. Vallejo, José Fernández-Seara, Josefa Fernández, Luis Lugo

**Affiliations:** 1 Departamento de Física Aplicada, Facultade de Ciencias, Universidade de Vigo, E-36310 Vigo, Spain; m.evaalvarez@yahoo.es (E.Á.-R.); jvallejo@uvigo.es (J.P.V.); 2 Área de Máquinas e Motores Térmicos, Escola de Enxeñería Industrial, Universidade de Vigo, E-36310 Vigo, Spain; jseara@uvigo.es; 3 Grupo NaFoMat, Laboratorio de Propiedades Termofísicas, Departamento de Física Aplicada, Universidade de Santiago de Compostela, E-15782 Santiago de Compostela, Spain; josefa.fernandez@usc.es

**Keywords:** nanofluids, functionalized graphene nanoplatelets, commercial coolant, energy efficiency, wind energy, convection heat transfer coefficient, pressure drop

## Abstract

Convection heat transfer coefficients and pressure drops of four functionalized graphene nanoplatelet nanofluids based on the commercial coolant Havoline^®^ XLC Pre-mixed 50/50 were experimentally determined to assess its thermal performance. The potential heat transfer enhancement produced by nanofluids could play an important role in increasing the efficiency of cooling systems. Particularly in wind power, the increasing size of the wind turbines, up to 10 MW nowadays, requires sophisticated liquid cooling systems to keep the nominal temperature conditions and protect the components from temperature degradation and hazardous environment in off-shore wind parks. The effect of nanoadditive loading, temperature and Reynolds number in convection heat transfer coefficients and pressure drops is discussed. A dimensionless analysis of the results is carried out and empirical correlations for the Nusselt number and Darcy friction factor are proposed. A maximum enhancement in the convection heat transfer coefficient of 7% was found for the nanofluid with nanoadditive loading of 0.25 wt %. Contrarily, no enhancement was found for the nanofluids of higher functionalized graphene nanoplatelet mass fraction.

## 1. Introduction

Energy is one of the main resources for society nowadays. Climate change, fossil fuel reservoirs depletion and the growth of worldwide energy demand have been the motivators for the looking of more efficient ways of energy generation, storage and consumption.

Thermal processes play a major role in energy generation from both convectional fuels and renewable resources. Thermal cycles, involving different heat transfer processes, are essential to transform energy, eventually converted into electricity in power plants from fossil and nuclear fuels, biomass or thermal solar energy. Nevertheless, cooling processes of components involved in energy generation are also susceptible of an improve in its efficiency.

Given the importance of increasing the efficiency of heat transfer processes, several approaches have been investigated over the past years to enhance heat transfer such as vibration techniques, application of electric and magnetic fields or surface modification, among others. However, particle addition to working fluids has been explored for several years and has become more and more relevant since Choi et al. [1] suggested the use of nanoparticle dispersions, nanofluids, for heat transfer applications, taking advantage of the enhanced thermal conductivity of solids compared to the poor thermophysical properties of common working fluids.

Although early attempts with bigger solid particle additives were carried out prior to Choi et al. [1], serious issues due to poor stability and deposits of the particles were solved using nanosized additives instead. The nature of the nanoadditives has been a matter of debate and several materials have been tested (metals [2,3], oxides [4,5,6,7], nitrides [8] and carbon allotropes, among others) leading to different degrees of heat transfer enhancement. However, the research community has widely agreed in the significant improvement in thermal performance of carbon allotrope dispersions such as carbon nanotubes [9], graphite [10], graphene nanosheets or nanoplatelets [11,12,13] and nanodiamonds [14] nanofluids.

The thermal conductivity increase of nanofluids is the main responsible of their potential heat transfer improvement. Different mechanisms, which give an in-depth explanation to this thermal conductivity enhancement have been reported in the literature. Iacobazzi et al. [15] reviewed and discussed the effect of molecular-level layering, Brownian motion, ballistic phonon motion, clustering and Kapitza resistance, among others, in the thermal conductivity enhancement of nanofluids. Yu and Choi [16] developed a modified Maxwell model to account with the influence of a nanolayer of liquid surrounding nanoparticles, which contributes to a significant thermal conductivity increase for nanoparticle diameters smaller than 10 nm. For bigger particles, the effect of the nanolayer, which is meant to have a higher thermal conductivity than the bulk liquid, is negligible [15,16]. Moreover, the layering mechanism has been reported to have more effect in metal-based nanoparticles leading to higher thermal conductivities, compared to the oxides, as Milanese et al. [17] found for Cu and CuO nanofluids. Brownian particle motion in nanofluids has found to be size-dependent, therefore, dispersions with smaller nanoparticles show significantly increased thermal conductivity, since larger random motion occurs [18]. However, the Brownian motion of larger particles has an insignificant effect on thermal conductivity [15]. The same trend was found in the effect of ballistic phonon motion for big size nanoparticles [15]. Nanoparticle clustering has shown to improve the thermal conductivity of the dispersions [15,19,20] but this phenomenon may have a negative effect on the overall heat transfer performance due to deposits and nanoparticle-free regions in the fluid, especially at low nanoadditive loading [19]. The Kapitza, thermal interfacial resistance represents a barrier against heat transfer, particularly for small nanoparticle dispersions with large interfacial resistance, in which a high nanoadditive loading may reduce the overall heat transfer performance [20].

Applications of nanofluids in heat transfer range from transport, electronic cooling [21], industrial cooling [22,23], cooling systems in aerospace and defence [9] and, more interestingly, in renewable energy [22,24,25], on which this research focuses. Overheating of wind turbine components inside the nacelle is an increasingly concerning issue, since the wind turbines size and the number of offshore wind farms have grown in the past years. Unfortunate combination of demanding operation conditions and high ambient temperatures usually leads to overheating of the gearbox and the generator, decreasing the overall energy efficiency and even requiring a reduction in the power given by the wind turbine [26]. This situation has been regularly solved by air-to-air cooling systems, which force an airflow through the nacelle to cool the components. The cooling capacity of these systems is very limited and usually insufficient for high power wind turbines (up to 10 MW nowadays [27]). In addition, the durability of the components inside the nacelle may be jeopardized by the traditional cooling systems due to the dust, sand or other materials introduced by the forced air flow [25]. Therefore, a novel approach to solve this overheating problem is water-to-air cooling systems, which are more sophisticated and in which nanofluids can be implemented [25]. A more widespread application of nanofluids in renewable energy is in thermal solar systems [24,28,29]. In addition to the enhanced heat transfer, solar applications take also advantage of the improved optical properties of nanofluids [29].

In many of the applications mentioned above where cooling is involved, at industrial scale commercial coolants are the typical working fluids, essentially mixtures of glycols and water that contain some additional components as anticorrosive or colorant liquids. Several studies have been carried out in order to determine the thermal performance of nanofluids using different base fluids but scarce studies have focused on commercial working fluids. In the literature for cooling applications, car engine coolants were chosen to study the effect of the addition of nanoparticles in the thermal performance [30,31,32]. In this work, a commercial coolant was chosen as base fluid, Havoline^®^ XLC Pre-mixed 50/50, which is a mixture of ethylene glycol, water and other additives that has been matter of study in our previous research for other characterization aspects [29,33].

Among carbon allotropes, graphene was reported to present an extremely high thermal conductivity, up to 5000 W·m^−1^·K^−1^ [34] and to enhance heat transfer greatly, as recent studies have revealed. Ghozatloo et al. [11] tested low concentrated (0.05, 0.075 and 0.1 wt %) graphene nanosheet dispersions in deionised water using a shell and tube heat exchanger. A maximum enhancement of 35.6% in convection heat transfer coefficients was found for the dispersion with 0.1 wt % graphene concentration. Sadeghinezhad et al. [12] found bigger improvements in heat transfer of graphene nanoplatelets water-based nanofluids with low concentrations (0.025, 0.05, 0.075 and 0.1 wt %), under turbulent flow and high heat flux conditions. The enhancements in heat transfer coefficients regarding the base fluid ranged between 13 and 160%. Pressure drop was also monitored but just a maximum increase of 14.6% was found [12]. More recently, Solangi et al. [35] studied the thermal behaviour of different loaded propylene glycol-treated graphene nanoplatelet aqueous nanofluids (0.025, 0.05, 0.075 and 0.1 wt %) in a circular copper tube with thermocouples mounted in (or attached to) its outer surface. Maximum enhancement in convective heat transfer coefficient of 119% was found for the highest concentration in turbulent regime, with the friction factor increasing by a 14%. Amiri et al. [36] carried out an experimental determination of convection heat transfer coefficients and pressure drops of crumpled nitrogen-doped graphene nanosheet water-ethylene glycol nanofluids (0.001, 0.005 and 0.01 wt %) in a double tube heat exchanger. A maximum enhancement in the convection heat transfer coefficient of 83% was found with a rather low, 0.01 wt %, nanoadditive loading. Arzani et al. [37] analysed different dispersions of tetrahydrofurfuryl polyethylene glycol-treated graphene nanoplatelets (0.025, 0.05, 0.075 and 0.1 wt %) in water for an annular heat exchanger. These authors found maximum increases in the convection heat transfer coefficient of 25.6% for the 0.1 wt % nanofluid in turbulent regime with a pressure drop of up to 39.3%. Agromayor et al. [38] studied different loaded dispersions (0.25, 0.50, 0.75 and 1.0 wt %) of sulfonic acid-functionalized graphene nanoplatelet in water by means of a tube-in-tube heat exchanger. Maximum enhancements of convection heat transfer coefficients of 32% were found for the 0.50 wt % nanofluid in turbulent regime with pressure drop increasing by a 56.9% for this concentration. Pérez-Tavernier et al. [39] employed functionalized graphene nanoplatelets to nanoenhance a propylene glycol: water 30:70 wt % mixture. The results of their tests by means of a tube-in-tube heat exchanger showed enhancements in the heat transfer coefficient that reached 15.3% for the 0.50 wt % concentration, with pressure drop rises of up to 12.4% for the highest concentration. The huge variability of the results obtained in different studies highlights the necessity of carrying out more experimental test to get a more homogeneous map of the thermal performance of this nanofluid family. Furthermore, up to our knowledge, no experimental studies were developed on the heat transfer performance of nanoenhanced commercial industrial coolants by using graphene derivatives.

Thus, the purpose of this research is to study the thermal performance of graphene nanoplatelet dispersions in Havoline^®^ XLC Pre-mixed 50/50 + 0.125 wt % SDBS, a commercial coolant used in water-to-air cooling systems in high power wind turbines, with a small content of sodium dodecyl benzene sulphonate, a surfactant that was added to enhance stability. The base fluid, as well as four different mass concentrations of graphene nanoplatelets were tested, 0.25, 0.50, 0.75 and 1.0 wt %. Experimental tests to obtain convection coefficients and pressure drops were carried out. Dimensionless numbers were analysed and correlations for Nusselt number and Darcy's friction factor suggested in order to predict the thermal performance of the nanofluids under different conditions.

## 2. Materials and Methods

### 2.1. Materials

Four nanofluids were prepared by the two-step method described in previous works [33,38]. The dispersions consisted of polycarboxylate chemically modified graphene nanoplatelets, fGnP, from NanoInnova Technologies S.L. (Madrid, Spain) and the base fluid, Havoline^®^ XLC Pre-mixed 50/50, Hav/W 50/50, commercialized by Chevron Products UK Limited (London, United Kingdom), as well as the surfactant sodium dodecyl sulphonate, SDBS, from Sigma-Aldrich (St. Louis, MO, USA). The nanoadditive loadings chosen were 0.25, 0.50, 0.75 and 1.0 wt % while the concentration of SDBS was kept constant at 0.125 wt % in all the nanofluids.

The selected amount of SBDS is a result of previous stability analyses based on zeta potential measurements [29,33]. The rising concentration of this surfactant increases the zeta potential of the dispersions. It was selected the 0.125 wt % loading because it ensures the overcoming of the 30 mV absolute zeta potential threshold (sign of good stability [40] by modifying as little as possible the properties of the original nanofluids [29,33]. These results were checked for all the analysed samples and at temperatures from 298 to 343 K [33].

The thermophysical properties of the base fluid and nanofluids used in these data analyses were experimentally determined in a previous work [33]. Density (*ρ*), isobaric specific heat capacity (*c_p_*), thermal conductivity (*k*) and dynamic viscosity (*η*) were obtained by pycnometry, differential scanning calorimetry, transient hot wire technique and rotational rheometry, respectively, in the temperature range from 293.15 to 343.15 K. The declared expended uncertainty of these measurements (*k* = 2) was 0.1% for the first case and 3% for the three last. Enhancements for *k* reaching 7.3% and increases for *η* of up to 20% were described [33]. Table 1 shows the values for the thermophysical properties at 298.15 K, one of the test temperatures. On the other hand, the water thermophysical properties were obtained from REFPROP database [41].

### 2.2. Experimental Setup

The experimental facility in which the experiments were carried out was similar to that used in previous studies [38,39], where the layout can be found. It consists of three hydraulic circuits for heating water, cooling water and nanofluids, two heat exchangers and auxiliary devices such as hydraulic pumps, valves and deposits. However, the main component of the experimental facility is the tube-in-tube heat exchanger, on which the analysis of the nanofluid performance was focused. An insulator, ensuring the free arrangement of the devices, covers all the elements.

The tube-in-tube heat exchanger is composed of two concentric tubes being an indirect contact heat exchanger. Thus, the heat is transferred through the surface of the inner tube, made of stainless steel AISI 316L, which also separates the two fluids. Table 2 shows the dimensions of the tube-in-tube heat exchanger.

The flow in the heat exchanger is arranged in countercurrent, in order to make the heat exchange more efficient. Nanofluids flow through the inner tube, absorbing heat from the water, while the heating water flows throughout the annular tube. Insulation was added in the outer part of the heat exchanger to avoid heat losses to the atmosphere, ensuring that all the heat was transferred from the heating water to the nanofluid and simplifying the analysis.

The purpose of the heating water loop is to transfer heat to the nanofluids in the tube-in-tube exchanger. It is a closed loop consisting of a 0.025 m^3^ tank reservoir, a variable speed hydraulic pump and three electric resistors, two of which are adjustable power resistors.

The nanofluid loop is also a closed loop through which the studied fluid flows, absorbing heat from the heating water in the tube-in-tube heat exchanger horizontally positioned. It also consists of a 3 dm^3^ tank, a variable speed hydraulic pump and a plate heat exchanger that cools again the studied fluid by cooling water.

The cooling water loop is the component that removes the heat from the nanofluids in the plate heat exchanger, leading to the achievement of stationary conditions. It is an open loop connected to the water network. It consists of a tank of 30 dm^3^ to avoid effects of possible fluctuations of the water network in the experimental test, a single speed hydraulic pump and a needle valve.

The data acquisition system consists of two flowmeters, four thermocouples, a pressure drop sensor, a data acquisition card and a group of hardware devices. The four Pt-100 Class-A thermocouples (Design Instruments, Barcelona, Spain), 0.2 K uncertainty, were inserted in the inlet and outlet pipes of the tube-in-tube heat exchanger for both the heating water and the nanofluid loops, in direct contact with the fluids. Two flowmeters, SITRANS F M MAG 1100 and 3100 (Siemens A/S Flow Instruments, Sønderborg, Denmark), 0.2% uncertainty, monitored the volumetric flow rates of the nanofluid and heating water loop. The differential pressure drop sensor SITRANS P DS III (Siemens A/S Flow Instruments, Sønderborg, Denmark), 0.1% uncertainty, measures the pressure loss along the inner tube of the tube-in-tube heat exchanger. Finally, LabView software enables the usage of PIDs to adjust the power of the hydraulic pumps and resistors in order to keep stationary conditions for flow and temperature. This software also monitors the data live and stores them in files for post processing.

### 2.3. Methods

Convection coefficient and pressure drop tests were carried out fixing the mean temperature and the flow rates of the nanofluids and the heating water along the tube-in-tube heat exchanger. Three pairs of nanofluid/heating water temperatures were considered, keeping constant a temperature difference between both fluids of 15 K. The nanofluid flow rate was increased from 0.2 to 0.7 m^3^·h^−1^ every 0.1 m^3^·h^−1^. On the other hand, the heating water flow rate was kept constant at 0.8 m^3^·h^−1^ to ensure turbulent conditions in the annular section of the tube-in-tube heat exchanger and could apply the Gnielinsky correlations [42] for the obtainment of their convection coefficients. Table 3 shows the parameters of the different performed tests.

In order to keep stationary conditions, the data acquisition system worked over the adjustable devices through the PIDs. Once achieved, the measurement values were recorded by the data acquisition system over 270 s, gathering 100 samples.

### 2.4. Data Analysis

The hydraulic behaviour was analysed based on the pressure drops while the thermal behaviour of the nanofluids was assessed by determining the convection coefficients under different conditions. In addition, the dimensionless analysis, involving the Nusselt number and the Darcy friction factor, provided a further assessment of the thermal and hydraulic behaviour of the fluids of interest. Although pressure drop data were directly registered from the experiments, convection coefficients and dimensionless parameters were should be determined.

#### 2.4.1. Convection Coefficients

An expression based on the combination of the first law of thermodynamics, the Newton's law of cooling and Fourier's law for heat transfer, was derived to obtain the heat transfer coefficients from the volumetric flow rates and temperatures registered during the experimental test. The heat flow rate in a heat exchanger, $\dot{Q}$, is determined by:

(1) $$\dot{Q}=\frac{1}{R}\Delta \theta_{lm}$$

where *R* and $\Delta \theta_{lm}$ are the overall thermal resistance and the logarithmic mean temperature difference, respectively. $\Delta \theta_{lm}$ was calculated as a function of the inlet and outlet temperatures of the nanofluid (*T_in nf_* and *T_out nf_*) and the heating water (*T_in hw_* and *T_out hw_*) arranged in cross-flow through the heat exchanger, according to:

(2) $$\Delta \theta_{lm}=\;\frac{\left(T_{in\;hw}-\;T_{out\;nf}\right)-\left(T_{out\;hw}-\;T_{in\;nf}\right)}{Ln\left(\frac{T_{in\;hw}-\;T_{out\;nf}}{T_{out\;hw}-\;T_{in\;nf}}\right)}$$

and $\dot{Q}$ was calculated according to the first law of thermodynamics, considering a stationary energy balance in the annular space of the tube-in-tube heat exchanger, through which the heating water flows:

(3) $$\dot{Q}={\dot{Q}}_{nf}={\dot{Q}}_{hw}$$

(4) $${\dot{Q}}_{hw}=\rho_{hw}V_{hw}c_{p}{}_{hw}\left(T_{in\;hw}-\;T_{out\;hw}\right)$$

where *V* mean flow rate and the subscripts *nf* and *hw* stand for nanofluid and heating water, respectively.

**Figure 1:**
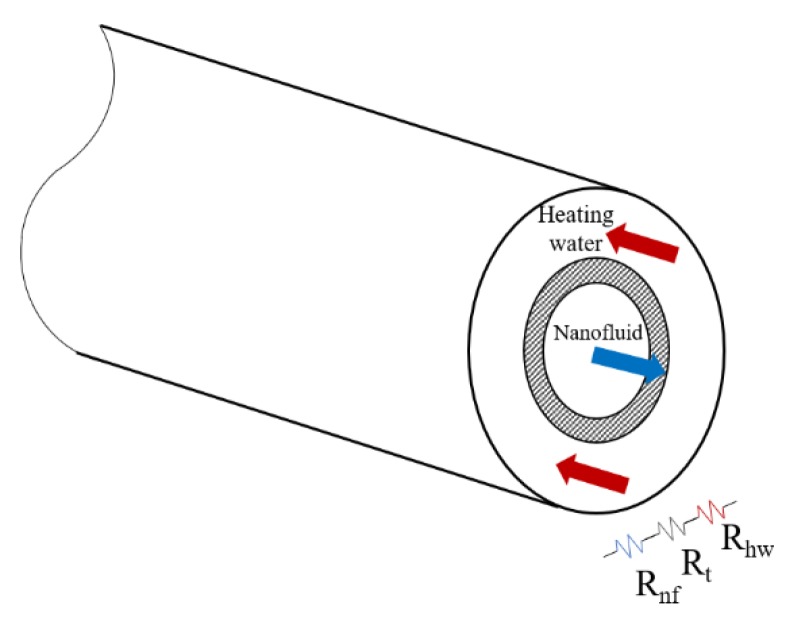
Thermal resistances in the tube-in-tube heat exchanger.

Figure 1 shows the distribution of the thermal resistances in the tube-in-tube heat exchanger. Since no heat losses to the atmosphere are assumed, the considered thermal resistances are three: the first due to convection in the heating water, *R_hw_*, the second due to conduction through the inner tube, *R_t_* and the third due to convection in the nanofluid, *R_nf_*.

Therefore, the overall thermal resistance *R* can be expressed as the sum of those mentioned above:

(5) $$R=R_{hw}+R_{t}+R_{nf}\;$$

Depending on the heat transfer mechanism, the thermal resistances were calculated in different ways. While *R_t_* was calculated according to Fourier's law and *R_hw_* and *R_nf_* were obtained from Newton's law of cooling:

(6) $$R_{t}=\;\frac{Ln\frac{d_{2}}{d_{1}}}{2\pi l_{h}k_{t}}\;$$

(7) $$R_{hw}=\;\frac{1}{h_{hw}\pi \left(d_{3}-d_{2}\right)l_{h}}\;$$

(8) $$R_{nf\;}\;=\;\frac{1}{h_{nf}\pi d_{1}l_{h}}$$

where *d*_1_, *d*_2_ and *d*_3_ are the inner diameter of the inner tube, the outer diameter of the inner tube and the inner diameter of the outer tube, respectively, *l_h_* is the effective length for heat exchange and *h* means convection heat transfer coefficient.

The heating water convection heat transfer coefficient *h_hw_* was calculated using the Gnielinski correlation [42] for fully developed turbulent flows in annular ducts. The boundary condition, outer wall insulated and heat flux only through the inner wall, was also taking into account, selecting the appropriate expression. Finally, the heat transfer coefficient of the nanofluids *h_nf_* was determined by combining Equation (1) with Equation (4) and finally substituting the expressions for the thermal resistances (Equations (6)–(8)).

#### 2.4.2. Dimensionless Analysis

The dimensionless analysis enables to assess the thermal performance of the nanofluids tested regardless the heat exchanger and system used for the experimental test and to extrapolate the results to any other application. The Nusselt (*Nu*), Reynolds (*Re*) and Prandtl (*Pr*) numbers and the Darcy friction factor (*f*) are key parameters for thermal characterization. Therefore, they were determined for nanofluids from the experimental data as follows:

(9) $$Nu_{nf}=\frac{h_{nf}d_{1}}{k_{nf}}$$

(10) $${Re}_{nf}=\frac{4\rho_{nf}V_{nf}}{\pi \eta_{nf}d_{1}}$$

(11) $${Pr}_{nf}=\frac{Cp_{nf}\eta_{nf}}{k_{nf}}$$

(12) $$f_{nf}=\frac{\pi^{2}d_{1}{}^{5}\Delta P_{nf}}{8l_{\Delta P}\rho_{nf}V_{nf}{}^{2}}$$

where *l*_Δ*p*_ is the effective length for pressure drop.

The uncertainty analysis of the measured and calculated properties was performed according to the procedures described by the Joint Committee for Guides in Metrology in the standard “Evaluation of measurement data—Guide to the expression of uncertainty in measurement (GUM)” [43].

In order to obtain a correlation for the Nusselt number, which may provide an estimation of the thermal behaviour of the nanofluids under different conditions than those in the experimental tests, an expression was suggested as function of *Re*, *Pr* and $\phi_{v}$, $Nu=\varphi \left(Re,Pr,\;\phi_{v}\right)K$, where a correction factor, *K*, was applied as Gnielinski [44] suggested:

(13) $$K={\left(\frac{Pr}{{Pr}_{s}}\right)}^{n}$$

where *n* is an index to be experimentally determined and *Pr_s_* is the Prandtl number obtained from the mean temperature of the heat transfer surface, in this case of the inner wall of the inner tube of the heat exchanger. This temperature is calculated according to:

(14) $$T_{s}=T_{m,nf}+R_{nf}\dot{Q}$$

Therefore, the suggested correlation can be expressed as follows:

(15) $$Nu=c_{1}{\left(1+100\;\phi_{v}\right)}^{c_{2}}{Re}^{c_{3}}{Pr}^{c_{4}}{\left(\frac{Pr}{{Pr}_{s}}\right)}^{c_{5}}$$

where *c*_1_ to *c*_6_ are the fitting parameters.

To assess the hydrodynamic behaviour of nanofluids the friction factor was analysed, together with the Reynolds number. Similarly, the correlation for the friction factor was suggested to depend on *Re* and $\phi_{v}$, $f=\varphi \left(Re,\phi_{v}\right)$.

The following equation based on empirical variables, *c*_6_ to *c*_8_, which are adjusted to the experimental data is proposed:

(16) $$f\;=\;c_{6}{\left(1+100\;\phi_{v}\right)}^{c_{7}}{Re}^{c_{8}}$$

In search of more efficient heat transfer fluids, the thermal behaviour, as well as the pressure losses must be taken into account. These two parameters should be simultaneously assessed since a fluid which produces high pressure drops may not appropriate even if it exhibits an improved thermal behaviour.

## 3. Results and Discussion

### 3.1. Validation

Firstly, validation tests were carried out using water at all the test conditions stablished for nanofluids, with the purpose of testing the experimental facility. The obtained convection heat transfer coefficients and pressure drops were compared with those predicted by Gnielinski correlations for pipe flows [44]. Gnielinsky correlations present validity ranges that are appropriate for this experimental study, 10^4^ < *Re* < 10^6^ and 0.1 < *Pr* < 1000. In addition, Gnielinski correlation for annular ducts was preferred over others such as Petukhov correlation because it accounts for the inner-to-outer diameter ratio of the annular tube in the friction factor empirical equation [42,45]. This ratio is meant to affect the velocity profile inside the annular section, thus influencing friction losses and heat transfer processes [45]. Gnielinski correlation for annular tubes has also been validated against more experimental data than others, showing low deviation [45].

**Figure 2:**
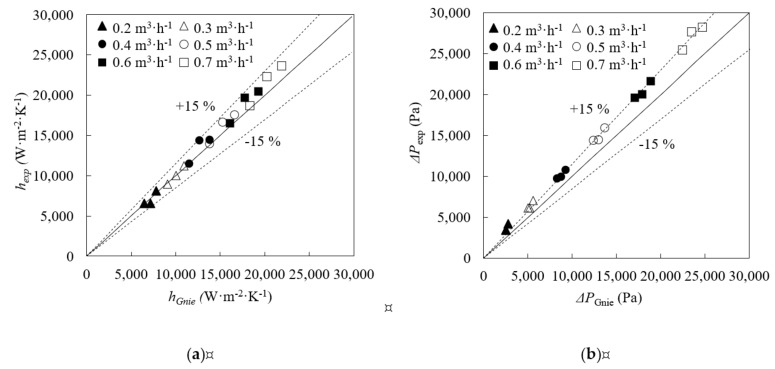
Comparison between experimental, exp and Gnielinski, Gnie, results for water. (**a**) Convection heat transfer coefficients, *h*. (**b**) Pressure drop, Δ*P*.

Experimental and Gnielinski convection heat transfer coefficients showed good agreement, Figure 2a, with an average deviation of 6.0% (most of the points fall within the dotted lines which indicates a deviation of 15%). The mean relative deviation for pressure drop is 13%, Figure 2b but it should be noticed that at low flow rates, the Reynolds number is at the lowest limit for Gnielinski correlation, 10,000.

No heat losses to the atmosphere were stablished in the data analysis. The average deviation between the heat absorbed by the water flowing by the inner tube and the heat transferred by the water flowing by the annular section achieves absolute deviations under 2.0% for all the test conditions, validating this assumption.

### 3.2. Heat Transfer Coefficients

**Figure 3:**
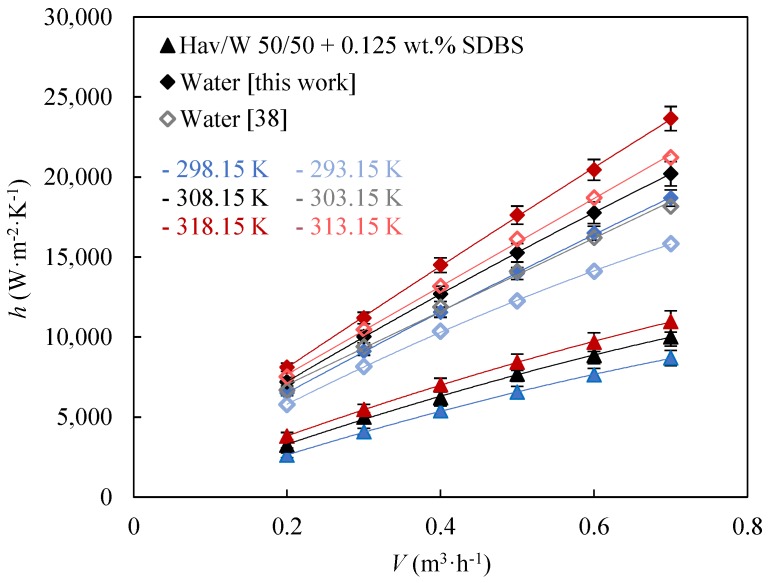
Experimental convection heat transfer coefficients, *h*, as a function of flow rate, *V*, for water and the base fluid at three temperatures. Error bars indicate the expanded uncertainty (*k* = 2). Literature convection heat transfer coefficients for water [38] are shown for comparison.

Figure 3 shows the experimental convection heat transfer coefficient as a function of the flow rate for water and for the base fluid, Hav/W 50/50 + 0.125 wt % SDBS, at three different temperatures. Literature convection heat transfer coefficients for water [38] are also shown for comparison. Water is one of the best heat transfer fluids, while mixtures of water with glycols, as in the case of the commercial coolant employed as base fluid, show lower thermal performance in exchange for a lower freezing point.

It should be noted that there is a good agreement between the heat transfer coefficients here presented for water and those previously determined [38]. Both fluids show higher convection heat transfer coefficients with increasing flow rate, since more turbulence enhances heat transfer and with increasing temperature, due to the rise in thermal conductivity and the decrease in viscosity [33].

**Figure 4:**
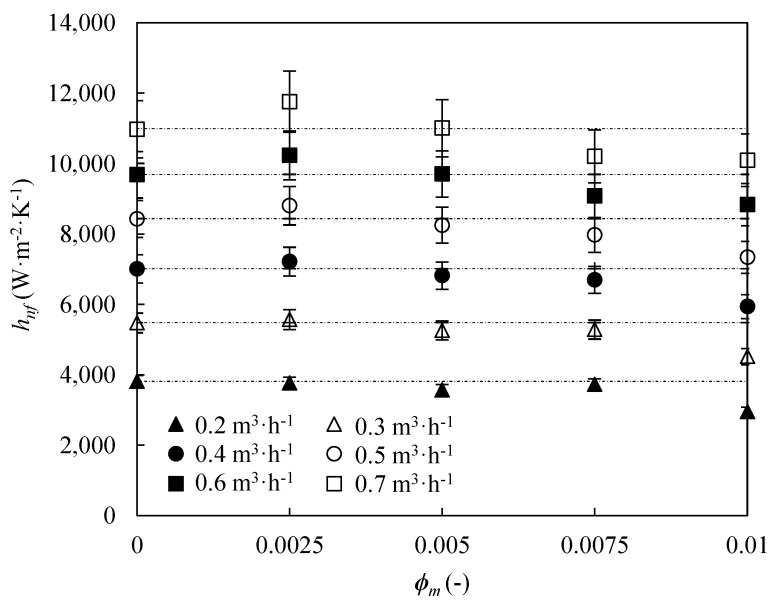
Experimental convection heat transfer coefficients, *h_nf_*, the nanoenhanced coolant as a function of fGnP mass fraction, *ϕ_m_*, for 6 different volumetric flow rates at *T_nf_* = 318.15 K. Error bars indicate the expanded uncertainty (*k* = 2).

Figure 4 shows the experimental convection heat transfer coefficients of the nanoenhanced coolant as a function of fGnP mass fraction for six different volumetric flow rates at *T_nf_* = 318.15 K. Higher convection heat transfer coefficients are obtained with increasing flow rates due to turbulence rise, as most of the literature works [46,47,48]. Several authors affirm that an increase in the turbulence intensity leads to better mixing within the nanofluid, leading to improvements in heat transfer [49,50].

On the other hand, 0.25 wt % nanofluid shows a clear improved convection heat transfer coefficient regarding the base fluid, particularly at high flow rates. Contrarily, higher contents of nanoplatelets seem to worsen the heat transfer. 1.0 wt % nanofluid shows clearly lower convection heat transfer coefficients than the base fluid, regardless the volumetric flow rate. Figure 4 displays that the optimum analysed nanoadditive mass fraction is 0.25 wt %. The same behaviour has been found at lower nanofluid temperatures. Different experimental studies in the literature have also shown the appearance of an optimum nanoadditive fraction that presents the maximum convection heat transfer increases [38,39,46,47,48,50,51,52,53,54]. Heris et al. [51] obtained the convection coefficients for different dispersions of alumina and cupric oxide in water, with nanoadditive loading ranging from 0.20 to 3.0 vol %, reaching the highest increases for 2.5 vol % of both nanoparticles. Farajollahi el al. [52] studied 0.30 to 2.0 vol % and 0.15 to 0.75 vol % concentrated dispersions of alumina and titania in water, respectively, obtaining maximum convection coefficient values with 0.50 vol % of alumina and 0.30 vol % of titania. Various nanoadditive loadings in the 0.20 to 2.0 vol % range of titanium dispersed in water were analysed by Duangthongsuk et al. [53], maximum enhancements being for 1.0 vol %. Azmi et al. [54] worked with 0.50 to 4.0 vol % nanoparticle concentrations of silica in water, achieving the highest increases for 3.0 vol %. Different nanoadditive concentration from 0.25 to 1.0 wt % of sulfonic acid-functionalized graphene nanoplatelets dispersed in water were analysed by Agromayor et al. [38], reaching the highest increases for 0.50 wt %. Pérez-Tavernier et al. [39] studied the 0.25 to 1.0 wt % concentrations of functionalized graphene nanoplatelets in propylene glycol:water 30:70 wt %, achieving a maximum enhancement in convection heat transfer coefficients for 0.5 wt %.

The dispersion of nanoparticles enhances the thermal conductivity of the base fluid but also increase its dynamic viscosity. The first effect should enhance the convection heat transfer because the heat exchange between particles inside the fluid takes place by conduction but the second tends to worsen it because of the intrinsic relation between viscosity and fluid flow. When the heat transfer coefficient increases, the first effect is greater than the second, while when the heat transfer coefficient decreases it occurs the contrary. The optimum nanoadditive loading is that for which the ratio between effects is the greater in favour of the first. For higher nanoadditive loadings, the viscosity increase effect over convection starts to be significant in relation to the thermal conductivity enhancement effect [39]. Moreover, Nikulin et al. [55] also argued that the nanofluid turbulence drops with increasing nanoadditive loading, effectively reducing the convection heat transfer coefficient of the nanofluids with highest fGnP mass fraction.

**Figure 5:**
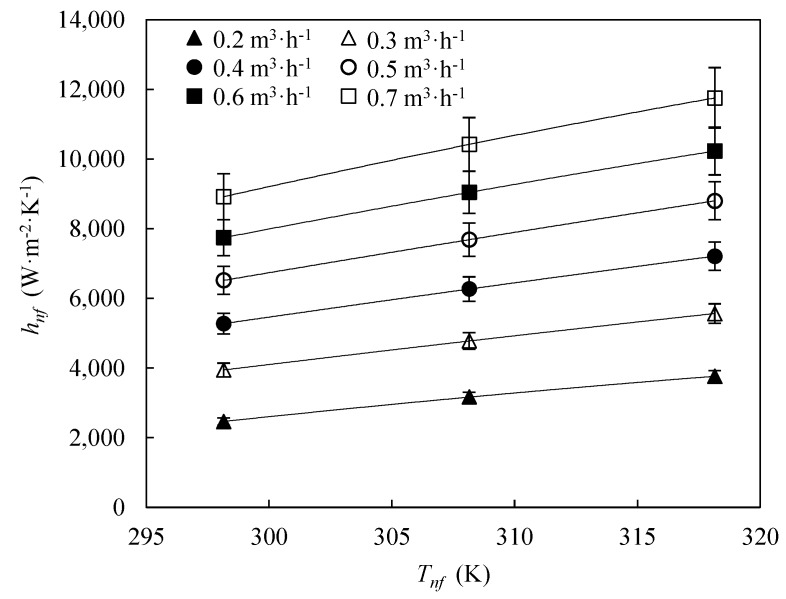
Temperature, *T_nf_*, dependence of convection heat transfer coefficients of 0.25 wt % nanofluid, *h_nf_*, for different volumetric flow rates. Error bars indicate the expanded uncertainty (*k* = 2).

Figure 5 shows the temperature-dependence of convection heat transfer coefficients of the experimental optimum concentration, 0.25 wt %, for different volumetric flow rates. Convection heat transfer coefficient rises with increasing temperature, regardless the volumetric flow rate. Higher temperature leads to an improved thermal conductivity [33] and a decreased viscosity [33], enhancing heat transfer.

**Figure 6:**
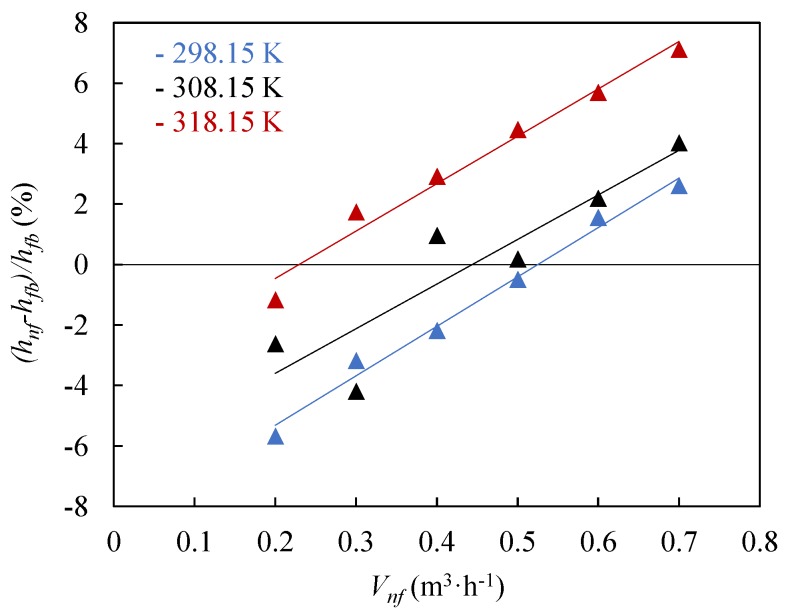
Enhancement of the convection heat transfer coefficient of 0.25 wt % nanofluid, *h_nf_*, regarding the base fluid, *h_bf_*, as function of the volumetric flow rate, *V_nf_*, for different temperatures.

Figure 6 shows the percentage enhancement of the convection heat transfer coefficients of the optimum nanofluid, 0.25 wt %, regarding the base fluid for different flow rates and temperatures. 

A maximum enhancement of 7.1% in the convection heat transfer coefficient of the 0.25 wt % nanofluid was found at *T_nf_* = 318.15 K, compared to the base fluid at the same condition. At lower temperature the enhancement in convection heat transfer coefficients is lower, achieving a maximum value of 2.1% at *T_nf_* = 298.15 K. Generally, the increase in convection heat transfer coefficients with the flow rate is almost linear. The maximum enhancements mentioned above were found for the highest flow rate, 0.7 m^3^·h^−1^.

### 3.3. Pressure Drops

**Figure 7:**
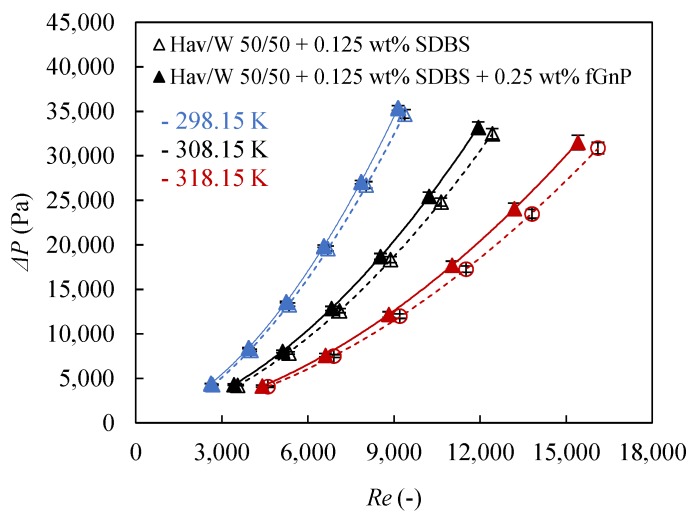
Reynolds number, *Re*, dependence on pressure drop, Δ*P*, for 0.25 wt % nanofluid and the base fluid at different temperatures. Error bars indicate the expanded uncertainty (*k* = 2).

Figure 7 shows the pressure drops as a function of the Reynolds number for the base fluid and the 0.25 wt % nanofluid at different temperatures. Pressure drop increases significantly with rising Reynolds number due to the increase of friction losses with turbulence for both fluids. For the same Reynolds number, pressure drops are smaller at high temperature, since the dynamic viscosity, the main property on which friction losses depends, reduces with increasing temperature.

Higher pressure drop indicates larger pumping power required in the system and this effect may be stronger than the convection heat transfer coefficient enhancement, reducing the overall system efficiency. Therefore, it is essential to take into account the pressure drop of the nanofluids regarding the base fluid, in order to compare the overall thermal performance. Figure 7 also shows slight increases in the pressure drop for the 0.25 wt % nanofluid regarding the base fluid for the same Reynolds number condition. However, the pressure drop trend of the 0.25 wt % nanofluid is very close to the base fluid trend, as Nikulin et al. [55] found in their study of isopropyl-Al_2_O_3_ nanofluids. While the maximum increase in pressure drops occurs at *T_nf_* = 318.15 K, around 14% for *Re* = 15,500, at lower temperature and Reynolds number the rise in pressure drop is smaller. The pressure drops found for the 0.25 wt % nanofluid seem to be slightly lower if compared with the results of Amiri et al. [36] who registered a maximum pressure drop enhancement around 6% for a graphene nanosheet dispersion of just 0.01 wt % in a base fluid similar to the industrial coolant used in this work. Therefore, the pumping power required by the optimum nanofluid should not be significantly larger than that required by the base fluid and the overall thermal performance should be improved almost in the same degree as the convection heat transfer coefficients, as shown in Figure 7.

### 3.4. Dimensionless Analysis

**Figure 8:**
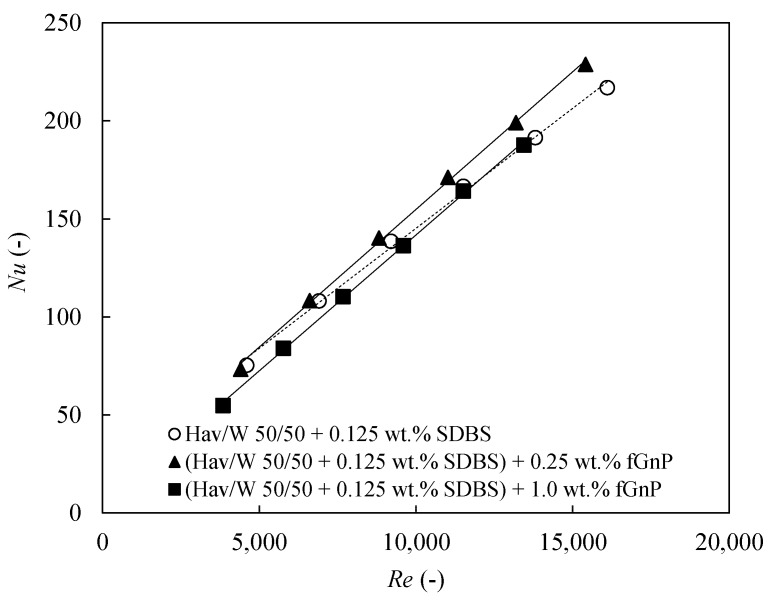
Nusselt number, *Nu*, as function of Reynolds number, *Re*, for base fluid and 0.25 and 1.0 wt % nanofluids at *T_nf_* = 318.15 K.

Figure 8 shows the Nusselt number as function of the Reynolds number for the base fluid and the 0.25 wt % and 1.0 wt % nanofluids at *T_nf_* = 318.15 K. Higher Nusselt numbers are achieved for increasing Reynolds number. As it was previously scrutinized, the higher level of turbulence improves the convection heat transfer coefficient. Thus, the increasing Reynolds leads to convection heat transfer coefficient increases while the thermal conductivity is maintained, explaining the Nusselt number enhancement. The 0.25 wt % nanofluid shows the best thermal performance of the nanofluids tested and the Nusselt number obtained are higher than those of the base fluid, particularly at high Reynolds number, getting a maximum Nusselt enhancement of 8.9% for *Re* = 15,500 and a minimum increase of 4.4% at *Re* = 4600, approximately. On the other hand, the 1.0 wt % nanofluid shows worse thermal behaviour than the base fluid for low turbulence regimes, while at higher Reynolds numbers their behaviour is similar.

As indicated in Section 2.3, empirical correlations are suggested for Nusselt number and friction factor from the experimental data, excluding that corresponding to 0.2 m^3^·h^−1^ since the low Reynolds number at this volumetric flow rate indicated that the flow was too close to the laminar regime. The following equation shows the proposed Nusselt number correlation, according to the form of the previous Equation (15):

(17) $$Nu=0.00758{\left(1+100\;\phi_{v}\right)}^{0.207}{Re}^{0.938}{Pr}^{0.402}{\left(\frac{Pr}{{Pr}_{s}}\right)}^{0.697}.$$

The mean deviation of this correlation from the experimental values is 3.7%, which is relatively low. The sign of the exponent of the term *Pr/Pr_s_* is positive because the nanofluid is absorbing heat. Therefore, this correlation is only valid for nanofluid absorbing heat and which parameters are within the ranges $0\%\;<\phi_{v}<0.47\%$, 3300<Re<16500, 23.8<Pr<33.9 and $1.05\;<\;Pr/{Pr}_{s}<\;1.24$.

Similarly, the following equation indicates the correlation for the friction factor, with the form previously suggested in Equation (16).

(18) $$f=0.466{\left(1+100\;\phi_{v}\right)}^{0.0295}{Re}^{-0.299}.$$

The mean deviation between the correlated and the experimental values is around 3.5% for the following validity ranges, $0\%\;<\phi_{v}<0.47\%$, 3300<Re<16500.

## 4. Conclusions

Convection heat transfer coefficients and pressure drops of a nanoenhanced industrial coolant at five different concentrations of functionalized graphene nanoplatelets (0, 0.25, 0.50, 0.75 and 1 wt %) were experimentally obtained. 

The analysis of the convection heat transfer coefficients shows that the 0.25 wt % nanofluid has the highest value of all the nanofluids tested and the base fluid, particularly at high temperatures and flow rates. The maximum enhancement in convection heat transfer coefficients for this nanofluid is over 7% regarding the base fluid. This nanofluid also shows the highest Nusselt number, indicating the best thermal performance among the fluids of study, regardless the physical device. Higher nanoadditive loading shows a drop in the convection heat transfer coefficient. This is explained by the rise in dynamic viscosity with increasing fGnP mass fraction, which penalizes heat transfer. The optimum nanofluid, Hav/W 50/50 + 0.125 wt % SDBS + 0.25 wt % fGnP, shows a small rise in pressure drops compared to the base fluid, thus the pumping power required with the 0.25 wt % fGnP nanofluid will probably remain the same as with the base fluid.

The empirical correlations for the Nusselt number and friction factor proposed show a deviation with the experimental values below 4%. Therefore, the correlations show a good reliability for the validity ranges given.

In conclusion, Hav/W 50/50 + 0.125 wt % SDBS + 0.25 wt % fGnP show an enhanced thermal performance regarding the base fluid in the experimental facility and a potential improvement in heat transfer in the cooling systems of wind turbines.

## Figures and Tables

**Table 1:** Density (*ρ*), isobaric specific heat capacity (*c_p_*), thermal conductivity (*k*) and dynamic viscosity (*η*) for the different loaded (Hav/W 50/50 + 0.125 wt % SDBS) + fGnP nanofluids at 298.15 K [33].

Thermophysical Property	Hav/W 50/50 + 0.125 wt % SDBS	0.25 wt % Nanofluid	0.50 wt % Nanofluid	0.75 wt % Nanofluid	1.0 wt % Nanofluid
*ρ* (kg·m^−3^)	1068.1	1068.9	1070.1	1071.1	1072.1
*c_p_* (J·kg^−1^·K^−1^)	3302	3296	3290	3284	3278
*k* (W·m^−1^·K^−1^)	0.390	0.397	0.405	0.412	0.418
*η* (mPa·s)	3.53	3.62	3.67	3.75	3.88

**Table 2:** Dimensions of the tube-in-tube heat exchanger.

Parameter	Nomenclature	Value (10^3^ m)
Inner diameter of the inner tube	*d*_1_	8
Outer diameter of the inner tube	*d*_2_	10
Inner diameter of the outer tube	*d*_3_	15
Effective length for heat exchange	*l_h_*	930
Effective length for pressure drop	*l*_Δ*P*_	1180

**Table 3:** Nanofluid temperatures (*T_m nf_*), nanofluid flow rates (*V_nf_*) and heating water flow rates (*V_hw_*) for the convection coefficient and pressure drop tests.

*T_m nf_* (K)	*V_nf_* (m^3^·h^−1^)	*V_hw_* (m^3^·h^−1^)
298.15	0.2–0.7	0.8
308.15	0.2–0.7	0.8
318.15	0.2–0.7	0.8

## Abbreviations

**Nomenclature**	
*c*_1_–*c*_8_	Correlation fitting constants
*c_p_*	Isobaric specific heat capacity, J·kg^−1^·K
*d*_1_	Inner diameter of the inner tube of the tube-in-tube heat exchanger, m
*d*_2_	Outer diameter of the inner tube of the tube-in-tube heat exchanger, m
*d*_3_	Inner diameter of the outer tube of the tube-in-tube heat exchanger, m
*f*	Darcy friction factor
fGnP	Functionalized graphene nanoplatelets
*h*	Convection heat transfer coefficient, W·m^−2^·K^−1^
Hav/W 50/50	Havoline^®^ XLC Pre-mixed 50/50
*k*	Thermal conductivity, W·m^−1^·K^−1^
*l_h_*	Effective length for heat exchange, m
*l*_Δ*p*_	Effective length for pressure drop, m
*Nu*	Nusselt number, dimensionless
*Pr*	Prandtl number, dimensionless
$\dot{Q}$	Heat flow rate, W
*R*	Thermal resistance, K·W^−1^
*Re*	Reynolds number, dimensionless
SDBS	Sodium dodecyl benzene sulphonate
*T*	Temperature, K
*V*	Volumetric flow rate, m^3^·s^−1^
Vol %	Nanoadditive volume concentration, %
wt %	Nanoadditive mass concentration, %
Δ*P*	Pressure drop along the tube-in-tube heat exchanger, Pa
Δ*θ_lm_*	Logarithmic mean temperature difference, K
*ρ*	Density, kg·m^−3^
*η*	Dynamic viscosity, Pa·s
*ϕ_m_*	Nanoadditive mass fraction, dimensionless
**Subscripts**	
*cw*	Cooling water
*hw*	Heating water
*in*	Inlet
*m*	Mean
*nf*	Nanofluid
*out*	Outlet
*t*	Inner tube of the tube-in-tube heat exchanger
